# Camera-based objective measures of Parkinson’s disease gait features

**DOI:** 10.1186/s13104-021-05744-z

**Published:** 2021-08-26

**Authors:** Jannis van Kersbergen, Karen Otte, Nienke M. de Vries, Bastiaan R. Bloem, Hanna M. Röhling, Sebastian Mansow-Model, Nicolien M. van der Kolk, Sebastiaan Overeem, Svitlana Zinger, Merel M. van Gilst

**Affiliations:** 1 Eindhoven University of Technology, 5612 AJ Eindhoven, The Netherlands; 2 Department of Neurology, Radboud University Medical Center, Geert Grooteplein Zuid 10, 6525 GA Nijmegen, The Netherlands; 3 Motognosis GmbH, Schönhauser Allee 177, 10119 Berlin, Germany; 4 Sleep Medicine Center Kempenhaeghe, Sterkselseweg 65, 5591 VE Heeze, The Netherlands; 5 NeuroCure Clinical Research Center, Charité – Universitätsmedizin Berlin, Corporate Member of Freie Universität Berlin, Humboldt-Universität zu Berlin, and Berlin Institute of Health, Berlin, Germany; 6 Experimental and Clinical Research Center, Charité - Universitätsmedizin Berlin Corporate Member of Freie Universität Berlin, Humboldt-Universität zu Berlin, and Berlin Institute of Health and Max Delbrück Center for Molecular Medicine, Berlin, Germany

**Keywords:** Parkinson’s disease, Movement disorders, Kinect TUG-test

## Abstract

**Objective:**

Parkinson’s disease is a common, age-related, neurodegenerative disease, affecting gait and other motor functions. Technological developments in consumer imaging are starting to provide high-quality, affordable tools for home-based diagnosis and monitoring. This pilot study aims to investigate whether a consumer depth camera can capture changes in gait features of Parkinson’s patients. The dataset consisted of 19 patients (tested in both a practically defined OFF phase and ON phase) and 8 controls, who performed the “Timed-Up-and-Go” test multiple times while being recorded with the Microsoft Kinect V2 sensor. Camera-derived features were step length, average walking speed and mediolateral sway. Motor signs were assessed clinically using the Movement Disorder Society Unified Parkinson’s Disease Rating Scale.

**Results:**

We found significant group differences between patients and controls for step length and average walking speed, showing the ability to detect Parkinson’s features. However, there were no differences between the ON and OFF medication state, so further developments are needed to allow for detection of small intra-individual changes in symptom severity.

## Introduction

Parkinson’s disease (PD) is the second most common age-related neurodegenerative disease, which markedly affects patients’ motor as well as a variety of non-motor functions. Various gait features are indicators of PD severity, such as a reduced step length, walking speed [1–4] and movement in the transverse plane [5, 6]. The disease severity can be scored clinically using standardized assessments, such as the Movement Disorder Society Unified Parkinson’s Disease Rating Scale (MDS-UPDRS) [7]. Administering this scale is time consuming and resource intensive for both patients and clinicians and is also susceptible to intra- and inter-rater variability due to the subjective nature of clinical scoring [8].

**Fig. 1:**
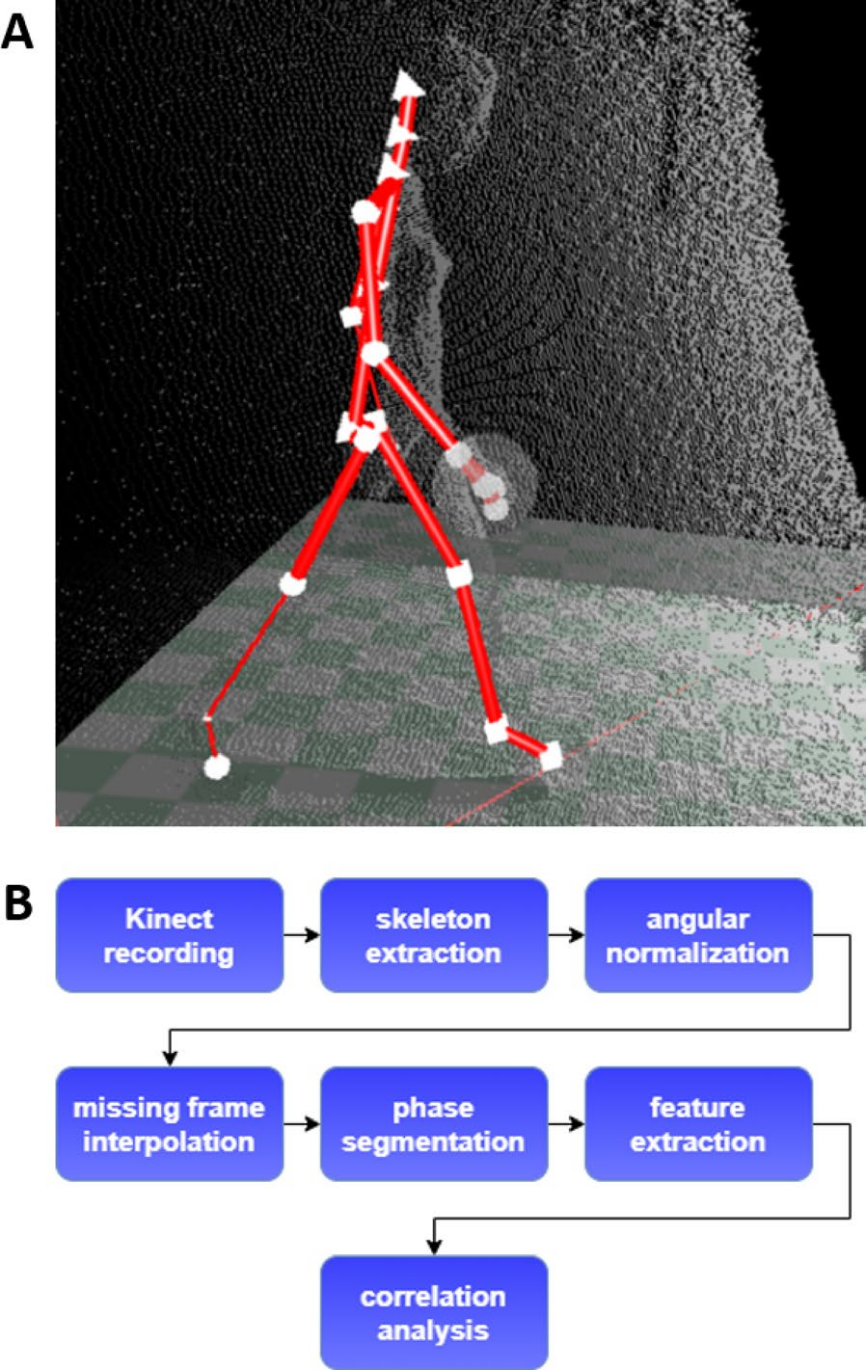
Landmark data & data analysis pipeline. **A** Example of a landmark data frame of a walking participant. Landmarks are represented in white. **B** Complete data analysis pipeline from data acquisition until correlation analysis of gait features and MDS-UPDRS-III score

Gait features can be measured objectively with 3D camera recordings. One promising low-cost consumer device is the Microsoft Kinect for Xbox One sensor (V2), which records RGB-depth data and tracks 25 anatomical landmarks in 3D space without the need for body-attached sensors. For an example, see Fig. 1A. Its accuracy and consistency have been validated with a gold standard system of optical motion capture (Vicon) [9, 10]. In recent studies, Kinect derived kinematic features were used to detect group-level differences of motor patterns between healthy controls and persons with PD for fine motor tasks such as finger tapping [11] and gait tasks [12, 13], including the “Timed Up-and-Go” (TUG) test [14]. However, these studies did not look at symptom severity changes within individuals with PD.

Detection of variation in individual symptom severity at home, could be a useful aid in monitoring disease progression and treatment effectiveness, giving clinicians time to focus on other aspects of patient care. Especially in times of a pandemic, benefits of home-based assessments become increasingly evident [15]. This would be especially valuable for persons with PD since considerable symptom fluctuations can be present, depending on factors such as the efficacy of medication, stress, fatigue or anxiety [16]. This study aims to investigate to what extent kinematic gait parameters, as derived with the Kinect, reflect intra-individual motor function differences ON and OFF medication, while performing a standard sequential motor task (TUG-test).

## Main text

### Methods

#### Participants

Data was collected in participants of the Park in Shape study [17], a randomized controlled trial evaluating the effectiveness of aerobic exercise on PD symptoms. The subjects mean age was 59.17 ± 8.22 years (SD) ranging 30 to 75 years. Subjects had idiopathic PD diagnosed by a neurologist, with Hoehn and Yahr (H&Y) stages I–II (tested off medication), no cognitive impairment (Mini-Mental State Examination > 24) and were stable on dopaminergic medication dose for at least 1 month. Exclusion criteria and a detailed design of the Park in Shape study can be found in the published protocol [17].

Healthy controls were recruited at the Radboud University Medical Center. The mean age was 34.57 ± 12.18 years (SD) with a range of 21 to 57 years. Exclusion criteria were neurodegenerative diseases or any other disease affecting motor function.

#### Data collection

*Study procedure* Participants abstained from taking their regular PD medication for 12 h before the first assessment (i.e. a practically defined OFF medication state). The motor symptoms were assessed using the MDS-UPDRS-III and participants performed the TUG-test three times. Then, participants took their regular medication and repeated both the MDS-UPDRS-III and the TUG-test after 90 min (ON medication state).

*Timed up-and-go test* The TUG-test is a standard movement task to assess balance and mobility [18]. The task consists of five sequential motor sub-tasks: standing up from a seated position, standing up, walking three meters forward as fast as possible, turning around and walking back to sit down again.

*Data acquisition* Test performance was recorded with the Kinect V2 sensor (Microsoft, Redmond WA, USA), which is able to record RGB-depth data and track 25 joint positions of persons in frame with a framerate of 30 Hz within a range of 0.5m up to 4.5m with a depth resolution of 512 × 424 pixels and a field of view of 70.6$^{\circ }$ × 60$^{\circ }$. The Kinect Sensor was placed shortly after the turning point of the TUG-test at roughly the participant’s eye-level.

#### Data analysis

A flowchart of the data analysis pipeline is displayed in Fig. 1B. For this pilot study only the first walking phase of the TUG-test was used, since its signal to noise ratio was the highest. Both the stand-up and sit-down phase were at the edge of the Kinect sensor range and therefore more noise corrupted. The turning phase occurred at the other edge of the sensor range, where the landmark tracking especially for the legs appeared to be unreliable. The second walking phase was also excluded, since the Kinect landmark tracking assumes that a person faces the camera, leading to less precise estimations.

*Data processing* From each recording, we extracted the 3D landmark time-series using a software tool built and provided by Motognosis based on the Kinect software development kit [19].

To avoid confounding from differing camera positions, we normalized landmark data with respect to camera roll and pitch.

Landmark signals from missing frames were interpolated linearly.

In order to cleanly extract the first walking phase, we segmented recordings according to the following procedure. The walking phase is characterized by periodic increase and decrease of the anterior-posterior distance between the feet. Therefore, we defined the start of the walking phase as the frame, in which the distance between the ankles exceeded a threshold of 0.1 meters for the first time.

The walking phase ends, as the participants starts to turn around. Therefore we defined it as the frame in which the horizontal projection of the shoulder distance starts to decline strongly. This decline is best seen as a negative peak in the second derivative (acceleration) of the shoulder distance.

#### Features

We used three gait features: walking speed, step length and mediolateral sway. Features were extracted using the Motognosis Labs software (Motognosis GmbH, Berlin), which was validated previously [9, 19] and averaged over all recordings of an individual per medication state. Additionally feature validation was done by visual inspection and outlier detection.

*Walking speed* The average walking speed was calculated by computing the time of participants walking phase and computing the Euclidean distance of the base of the spine from the first to the last frame.

*Step length* The step length is defined as the distance between the ankle landmarks, while both feet are on the ground. It was extracted with the Motognosis Labs software by finding the stance phases for both feet. To increase stance phase accuracy a velocity threshold was introduced and additional step length thresholds were implemented to filter out outliers.

*Mediolateral sway* We extracted the mediolateral sway by calculating the standard deviation of the mediolateral movement of the base of the spine.

#### Statistical analysis

To investigate the correlation between the MDS-UPDRS-III score and the gait features, we calculated Spearman’s rank correlation coefficient for both the ON and OFF condition. Furthermore, we used t-tests (paired tests in case of patients in ON and OFF condition) to investigate the group differences per extracted feature. We calculated the effect size of group differences as Cohen’s *d*. The statistical tests were not corrected for multiple comparisons as the study was explorative and descriptive in nature. Additionally the group difference in MDS-UPDRS-III score was calculated to ensure that disease severity differed significantly between the ON and OFF state.

### Results

#### Data

We included 19 persons with PD and 8 healthy controls.

Of the 145 total recordings, 128 recordings were used for analysis. We excluded recordings if the landmark data could not be extracted, the participant ran during the walking phase or if the participant had an atypical response to the medication, where the MDS-UPDRS-III score increased after taking medication. More recordings from TUG tests performed in ON state than in OFF state had to be excluded leading to the following data distribution:

- PD OFF: 60 recordings from 18 persons
- PD ON: 44 recordings from 12 persons
- control 24 recordings from 8 persons

#### Main results

The main findings are displayed in Table 1. The MDS-UPDRS-III group difference between ON and OFF group was significant (ON: 24.5 ± 14.4 vs. OFF: 34.9 ± 15.5, p < 0.0001), indicating a clinical discernible medication effect. There were significant group differences between PD patients (both ON and OFF) and controls, for the features step length and walking speed with effect sizes larger than 1 for all comparisons. Within the PD group, intra-individual differences and gait parameters between the ON and OFF state were not statistically significant.

**Table 1:** Results

Metric	Step length (m)	Avg. speed (m/s)	Mediolateral sway (cm)
Mean HC (SD)	0.776 (0.091)	1.357 (0.218)	1.544 (0.690)
Mean OFF (SD)	0.613 (0.102)	1.024 (0.192)	1.692 (0.526)
Mean ON (SD)	0.664 (0.118)	1.005 (0.186)	1.787 (0.610)
Difference OFF-HC (rel. diff.)	-0.16 (5.84%)	− 0.333 (6.98%)	0.148 (2.29%)
Difference ON-HC (rel. diff.)	− 0.111 (3.87%)	− 0.351 (7.44%)	0.243 (3.65%)
Difference OFF-ON (rel. diff.)	− 0.051 (2.00%)	0.019 (0.46%)	− 0.095 (1.36%)
p-value of t-test OFF-HC (Cohen’s d)	0.001 (− 1.643)	< 0.001(− 1.665)	0.552 (0.256)
p-value of t-test ON-HC (Cohen’s d)	0.047 (− 1.004)	0.001 (-1.797)	0.418 (0.383)
p-value of t-test OFF-ON (Cohen’s d)	0.218 (− 0.461)	0.792 (0.010)	0.654 (− 0.166)
Corr. coef. OFF with UPDRS (p-value)	0.08 (0.77)	0.01 (0.97)	0.599 (0.01)
Corr. coef. ON with UPDRS (p-value)	− 0.530 (0.09)	− 0.260 (0.44)	0.361 (0.28)

For associations between extracted features and disease severity, only the correlation of the mediolateral sway with the MDS-UPDRS-III score in the OFF group was significant (r = 0.599, p = 0.01).

**Fig. 2:**
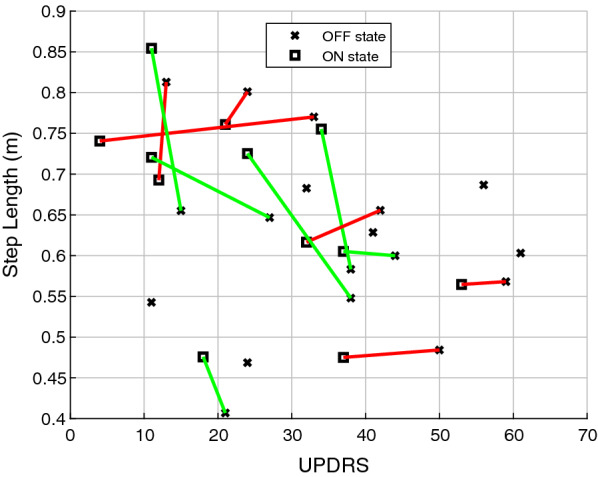
Comparison of step length parameter in ON and OFF state, lines connect measurements in ON and OFF state. Red lines represent a decreased step length after taking medication, green lines represent an increased step length

Fig. 2 shows the MDS-UPDRS-III score plotted against the step length for participants in ON and OFF state. For six of the twelve participants (with available ON and OFF recordings), step length increased after taking medication.

### Discussion

In our sample, the Kinect Sensor in combination with Motognosis Labs software was capable of extracting several gait features in PD patients during a functional mobility test. There were significant differences in step length and average speed between patients with PD and controls, with a large effect size (Cohen’s $d>1$). This confirms that the Kinect sensor is a promising tool to quantify PD-specific gait behaviour [2, 4, 6]. However, although a medication effect could be detected clinically, there were no significant differences in task features between the OFF and ON conditions.

This suggests that the current setup is not sufficiently sensitive to relatively small differences in motor functioning between the ON and OFF condition in persons with mild symptoms (only H&Y stages I and II included). However, in almost all participants the step length either increased or did not change strongly in the ON condition, indicating a trend towards an increased step length for reduced MDS-UPDRS-III scores, which is in line with previous research [1, 2, 14]. Interestingly, the strongest correlation with MDS-UPDRS-III was found for mediolateral sway. This was not expected, since Parkinsonian gait is characterized by stiffness and decreased transversal motion. A possible explanation might be the compromised balance of people with PD [20, 21] resulting in more compensatory movements.

The current study may well have been underpowered to detect differences intraindividual gait differences in ON versus OFF. We should also point out that we included patients with relatively mild disease, where the differences between the ON and the OFF state, although clinically evident, were not very large. Possibly some of these differences were caused by symptoms that are not directly reflected in gait. Much larger differences are typically seen in more advanced patients who experience response fluctuations, and it is possible that quantitative gait analysis using the remote kinematic analysis can detect relevant differences in such a more severely affected population. However, the practical use of these camera systems would mainly lie in ambulatory follow-up of disease progression or medication effect, necessitating sufficient sensitivity on an individual level. Since PD is a complex disease with a very heterogeneous presentation, increasing the number of outcome parameters and combining them in a single model may enable better detection of intra-individual differences. Using an adapted version of the TUG, such that the whole sequence can be successfully parametrized, or additional instrumentally assessed, standardized motor tasks such as the Short Physical Performance Battery [22] could expand on the kinematic features. Because the hypothesized intra-individual differences are likely small, standardized movement tests have an advantage over free movement by producing comparable, clean data and forcing a range of different movement patterns.

### Conclusion

We were able to detect group differences in gait features between people with PD and healthy controls using the Kinect depth camera. However, the current task setup and analysis approach lacks sensitivity to detect small intra-individual changes in symptom severity.

## Limitations

Limitations of this study include the small sample size, subjects with relatively mild symptoms and a not complete age match with the control population. The standard outcome for the TUG (task duration) could not be analysed because of missing frames at the beginning of the recording.

## Data Availability

The datasets generated and analysed during the current study are available from the authors upon reasonable request. There are limitations with regard to raw RGB and depth data because of identifiability of patients.

## Abbreviations

PD: Parkinson’s disease
TUG: Timed Up-and-Go
MDS-UPDRS: Movement Disorders Society Unified Parkinson’s Disease Rating Scale
